# Effects of High-Intensity Interval Training With Specific Techniques on Jumping Ability and Change of Direction Speed in Karate Athletes: An Inter-individual Analysis

**DOI:** 10.3389/fphys.2021.769267

**Published:** 2021-11-18

**Authors:** Alex Ojeda-Aravena, Tomás Herrera-Valenzuela, Pablo Valdés-Badilla, Eduardo Báez-San Martín, Jorge Cancino-López, Jairo Azócar Gallardo, José Zapata-Bastías, José Manuel García-García

**Affiliations:** ^1^Laboratorio de Investigación del Movimiento Humano, Departamento de Ciencias de la Actividad Física, Universidad de Los Lagos, Puerto Montt, Chile; ^2^Laboratorio de Entrenamiento Deportivo, Facultad de Ciencias del Deporte, Universidad de Castilla-La Mancha, Toledo, Spain; ^3^Escuela de Ciencias de la Actividad Física, el Deporte y la Salud, Universidad de Santiago de Chile, Santiago, Chile; ^4^Departamento de Ciencias de la Actividad Física, Facultad de Ciencias de la Educación, Universidad Católica del Maule, Talca, Chile; ^5^Carrera de Entrenador Deportivo Escuela de Educación, Universidad Viña del Mar, Viña del Mar, Chile; ^6^Departamento de Deportes y Recreación, Facultad de Ciencias de la Actividad Física, Universidad de Playa Ancha, Valparaíso, Chile; ^7^Exercise Science Laboratory, Faculty of Medicine, School of Kinesiology, Universidad Finis Terrae, Santiago, Chile

**Keywords:** combat sports, martial arts, athletes, physical fitness, strength and conditioning

## Abstract

This study investigated the effect of 4weeks of high-intensity interval training (HIIT) with specific techniques and analyzed inter-individual variability [classified in responders (Rs) and non-responders (NRs)] on jumping ability and change of direction speed (CODS) in youth karate athletes. Athletes of both genders (*n*=10) were randomly assigned into experimental group (EG; *n*=5) and the control group (CG; *n*=5). The EG trained 2–3days per week applying HIIT (three rounds [15 sets of 4s all-out specific efforts with 8s of dynamical pauses] with 3min of recovery between rounds) during their usual training during 4weeks. Assessments included squat jump (SJ) and countermovement jump (CMJ) and CODS by T-test. No significant interaction effect group by time was found. Although, in percentage and effect size (ES) terms increases were reported in both groups for SJ (EG: 15.2%, ES=0.91 vs. CG: 12.4%, ES=0.02) and only in EG for the T-test (−1.7%; ES=−0.35). In turn, a trend toward a higher proportion of Rs was observed in the EG (40% Rs) vs. CG (20% Rs) for SJ and CODS, respectively. In conclusion, the addition to regular training of a HIIT with specific techniques and based on the temporal combat structure after 4weeks was not a sufficient stimulus to increase jumping ability and CODS in karate athletes.

## Introduction

Karate is a popular combat sport that officially debuted at the Tokyo 2020 Olympic Games and whose performance requires athletes to possess a specific physical and physiological profile and technical expertise of the discipline (Chaabene et al., 2012). The “kumite” or combat modality is described as an intermittent nature (average effort/pause ratio 10:16.2s or 1:1.5–1:2; Chaabene, 2015; Tabben et al., 2018) and high-intensity activity (>90%HRmax; La^−1^>7.7±1.9mmol/L). In terms of physical performance, during combat, the athletes must strike and/or kick applying force quickly and explosively to score (Tabben et al., 2018). Among the most commonly used techniques include punching techniques with upper (in form of straight attacks) and lower limbs (using e.g., circular kicks or “mawashi geri”; Chaabène et al., 2014; Chaabene, 2015; Tabben et al., 2018). In addition, they must move in multiple directions to evade and/or counterattack (Chaabène et al., 2014; Chaabene, 2015; Tabben et al., 2018).

Based on the above approach, coaches should incorporate effective training strategies to develop sport-related fitness. Among other physical abilities, include the dynamic strength characteristics such as muscle power and efficient use of the stretch-shortening cycle (Chaabene et al., 2012; Loturco et al., 2014; Quinzi et al., 2020). Particularly, the dynamic strength characteristics of lower limbs are assessed using different technologies (e.g., contact platform, smartphone, force platform, and isokinetic device) and metrics (e.g., rate of force development, muscle power, and one-repetition maximum Loturco et al., 2014; Margaritopoulos et al., 2015; Kavvoura et al., 2018; Kostikiadis et al., 2018; Quinzi et al., 2020). In addition, in karate, a specific systematic review (Chaabene et al., 2012) and correlational and explanatory studies use the squat jump (SJ) and countermovement jump (CMJ; Chaabene et al., 2012; Loturco et al., 2014; Chaabene, 2015). In this sense, international athletes exhibit higher SJ and CMJ height performance than amateur athletes (Chaabene et al., 2012). Furthermore, this ability has been shown to significantly influence the speed and acceleration of punching execution (Loturco et al., 2014; Quinzi et al., 2020). In turn, agility including change of direction speed (CODS) is proposed as another important physical ability in this sport (Chaabene et al., 2012; Herrera-Valenzuela et al., 2020). In this regard, recent evidence shows a significant relationship between CODS with jumping ability in junior and cadet elite level karate athletes (Herrera-Valenzuela et al., 2020), as well as being a predictor of competitive success (i.e., medalists in European championships) in female karate athletes (de Quel et al., 2020).

In this context, high-intensity interval training (HIIT) according to recent systematic reviews in combat sports reports shows improvements in athletes’ fitness (Franchini et al., 2019; Vasconcelos et al., 2020). In karate, HIIT studies include protocols based on repeated-CMJ (Ojeda-Aravena et al., 2019) and repeated-sprints (Ravier et al., 2009) after 6–7weeks on jumping ability, CODS (Ojeda-Aravena et al., 2019), aerobic (Ojeda-Aravena et al., 2019), and anaerobic (Ravier et al., 2009) components. In addition, recent reports have incorporated the inclusion of HIIT using specific techniques in combat sports such as taekwondo (Aravena et al., 2020; Ouergui et al., 2020, 2021; Ojeda-Aravena et al., 2021a) and boxing (Kamandulis et al., 2018; Herrera-Valenzuela et al., 2021). Among the relevant results, significant inconsistent increases in jump height and CODS performance are reported (Ouergui et al., 2020, 2021; Herrera-Valenzuela et al., 2021; Ojeda-Aravena et al., 2021b).

In addition to the above, it is relevant to indicate that studies usually report the outcomes in group form (i.e., the mean change within a training group), without considering the athletes inter-individual variability of the athletes after training. In this sense, this research topic has been the subject of study since the 1980s in precision medicine to find responders (Rs) and non-responders (NRs) to physical exercise treatment applied to sedentary and/or comorbid obese individuals and recently in the field of applied sports science to understand athlete responses (Bonafiglia et al., 2016; Güllich, 2018; Ramirez-Campillo et al., 2018; Pickering and Kiely, 2019; Schulhauser et al., 2020; Talsnes et al., 2020). Furthermore, in combat sports, to date, some reports include taekwondo (Ojeda-Aravena et al., 2021b) and boxing (Herrera-Valenzuela et al., 2021).

Consequently, the potential efficacy of HIIT with specific techniques on the group and inter-individual response on jumping ability and CODS performance in karate athletes could be useful to provide relevant information to coaches on training adaptation mechanisms and individualization in sports training programming. Therefore, this study investigated the effect of 4weeks of HIIT with specific techniques and analyzed inter-individual variability (classified in Rs and NRs) on jumping ability and CODS in youth karate athletes. The rationale for the hypothesis is based on the notion that the ecological specificity of HIIT (i.e., using a sport-specific time structure and modality) could develop greater adaptations than usual training.

## Materials and Methods

### Participants

**Figure 1:**
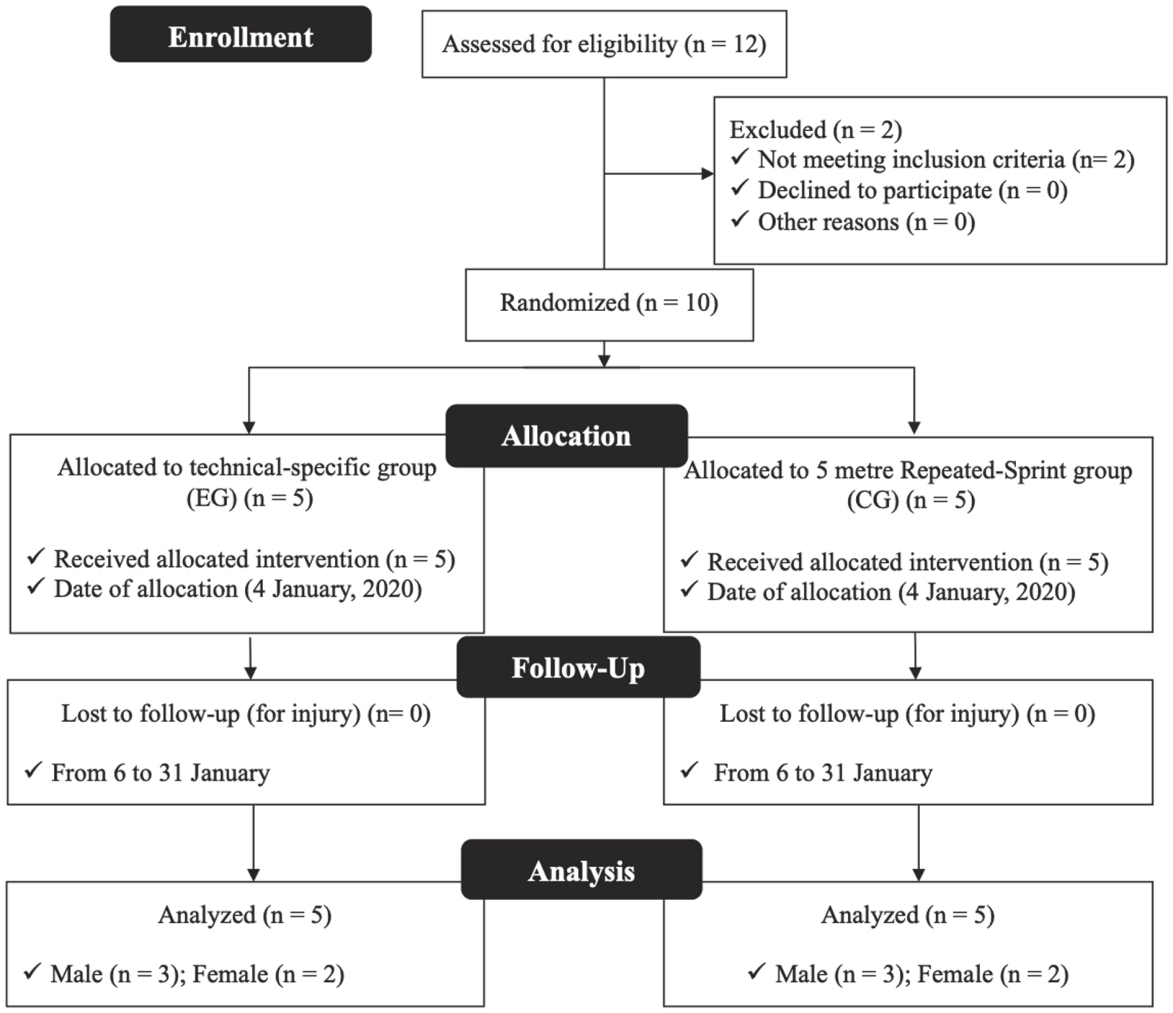
Flow chart of the process followed in the study.

Ten cadet karate athletes (age 15.2±1.6years; height 164.8±7.7cm; body mass 64.0±14.5kg) who compete annually in national and international level tournaments completed this study. They were invited to participate in the study during the annual planning transition period (January 2020) and randomly assigned into experimental group (EG; *n*=5; age 16.1±1.12years; height 168.8±7.6cm; body mass 68.5±20.9kg) and control group (CG; *n*=5; age 14.5±2.0years; height 160.8±7.1cm; body mass 59.6±6.5kg). Each group consisted of two females and three males (for details see Figure 1). To participate in the study, all athletes had to meet the following inclusion criteria (i) three years or more of karate experience; (ii) no history of disease and medication; (iii) no injuries or fractures during at least the last six months; (iv) consistently training at least three times per week for at least 6h per week; (v) membership in the National Karate Federation; (vi) not undergoing a period of body mass reduction; and (vii) participation in at least 85% of the intervention sessions. All athletes and/or family members of athletes under 18years of age were previously informed of the study purposes, associated benefits, experimental procedures, and potential by informed consent or informed assent before the assessments and training sessions. The study was conducted in compliance with the ethical standards for sport science studies (Harriss and Atkinson, 2015) and implemented after approval by the university ethics committee Autónoma university following the Helsinki declaration for work with humans (General Assembly of the World Medical Association, 2014).

### Assessments

#### Jumping Ability

Jumping ability was assessed by the SJ to assess concentric muscle actions and the CMJ to assess the slow stretch-shortening cycle or SSC through the maximum height reached (cm) using an electronic contact platform (Ergojump; Globus, Codogne, Italy; accuracy: 0.01m). For the SJ test, each athlete was previously instructed to place hands on hips, feet, and shoulders wide apart, and adopt a flexed-knee position (approximately 90°) for threes, and then perform a maximal effort vertical jump. Meanwhile, for the CMJ test, each athlete was previously instructed to rest hands on hips, feet, and shoulders well apart, and perform a downward movement (no restriction was placed on the knee angle achieved) followed by a vertical maximal effort (Ramirez-Campillo et al., 2013; Groeber et al., 2020). Intra-class correlation or ICC SJ pre=0.91 (CI 95% 0.80–0.96); ICC SJ post=0.90 (CI 95% 0.80–0.90), ICC CMJ pre=0.93 (CI 95% 0.90–0.98); and ICC CMJ post=0.95 (CI 95% 0.90–0.98).

#### Change of Direction Speed

The T-test was used to assess CODS during multidirectional movement (i.e., forward, lateral, and backward; Seo et al., 2019). For which four cones were set up in a “T” shape. Where the athlete started at a sound signal to run in a straight line to cone A, then ran at maximum speed to cone B (A – B: 5m) touching the top of the cone with the right hand; then, he turned left and ran away as fast as possible with lateral steps to cone C (B – C: 5m) until he touched the top of the cone. Then, he reversed directions and moved away using lateral steps to meet cone D (C – D: 10m) and touched the top of the cone. After that, he laterally stepped backward to touch cone B (D – B: 5m) and finally ran backward to cone A (B – A: 5m). Speed was recorded by an automatic timing system using electronic photocells (Brower Timing System, Salt Lake City, UT) accurate to 0.001s. The gates were positioned 1-m above the ground. ICC pre=0.90 (CI 95% 0.87 a 0.92) and ICC post=0.92 (CI 95% 0.90 a 0.96).

### Training Program

The training program had a duration of 10 sessions (4weeks) of 90min each session and was applied on 3 non-consecutive days (Monday, Wednesday, and Friday). The training load distribution was oriented to technical-tactical development with the coach’s permanent intervention during the training sessions. The HIIT was performed in front of a partner who did not participate in the study. Specifically, the protocol mimicked the official combat duration (3min). In addition, the HIIT intervals were based on the documented temporal structure for this sport (1:2; Tabben et al., 2018). Previously, both groups were instructed to use the rating of perceived exertion scale (RPE 0–10) to internal load control (Tabben et al., 2015; Slimani et al., 2017; Ouergui et al., 2020; Ojeda-Aravena et al., 2021a). The All-out HIIT format was used (Laursen and Buchheit, 2018). The training load was increased by decreasing the density volume during the last week, without modifying the high-intensity time. Briefly, the first 2weeks the athletes recovered in 3min between rounds and performed HIIT at a frequency of twice a week. In the last 2weeks, the density decreased to 2min of recovery and performed three times per week. Specifically, each training session started with a standardized 15-min warm-up group consisting of circle jogging (5min) and lower and upper body dynamic stretching (10min). Subsequently, the EG group was separated from the total group of athletes to execute the HIIT with specific techniques (∼20min; Laursen and Buchheit, 2018; Franchini, 2020). Particularly, athletes executed three rounds of 15 sets of 4s all-out efforts of straight punch and circular kick combinations in front of a partner followed by 8s of low intensity by performing a combat stance (imitating the combat stance). The striking sequence included an initial straight punch with the front hand or “oi tsuki,” followed by a circular kick “mawashi geri” with the back leg or “giaku mawashi geri,” a straight punch with the backhand or “giaku tsuki” and a kick with the front leg “oi mawashi geri” (Chaabene et al., 2015). In parallel, the CG continued with their usual training.

**Figure 2:**
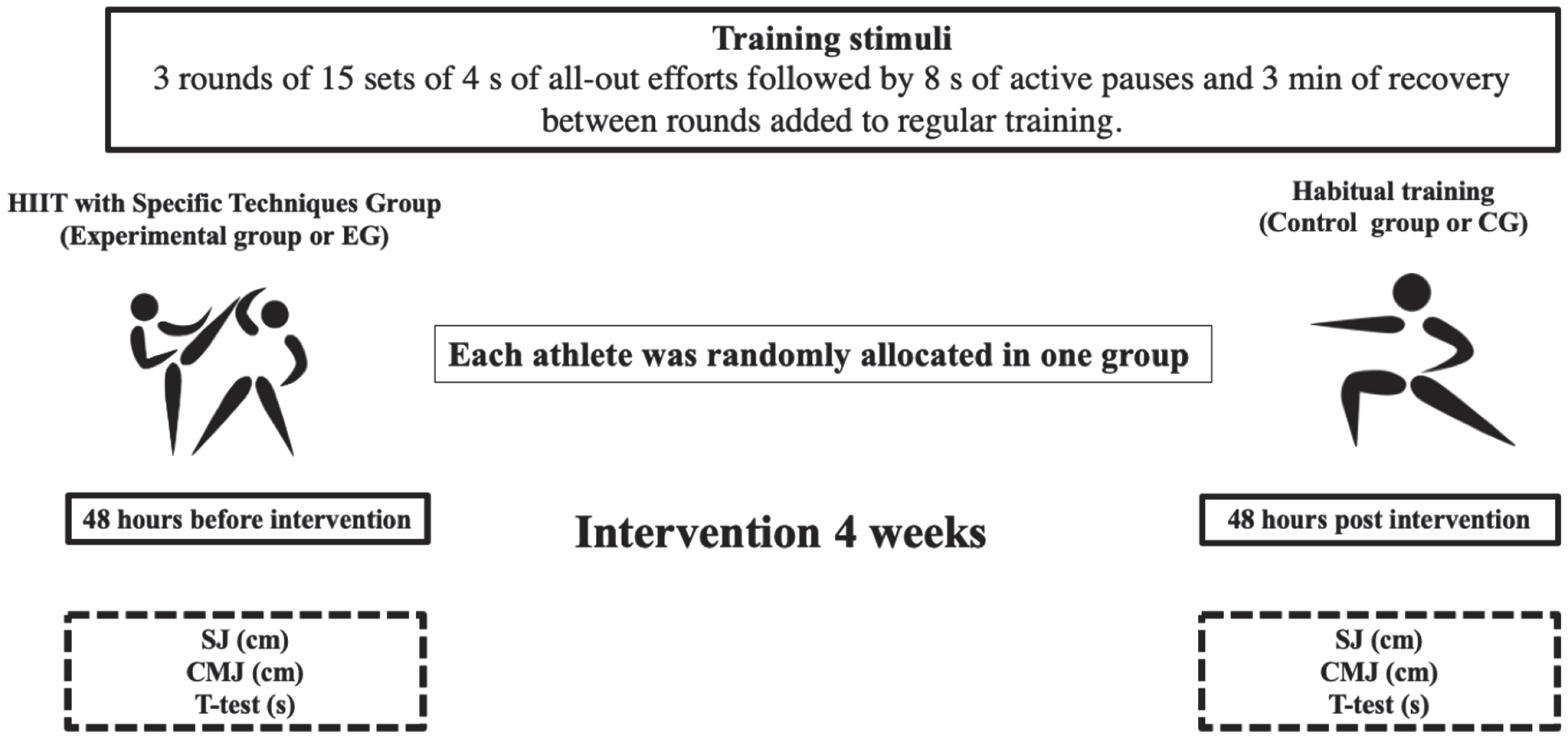
Graphic representation of the experimental design of the study. SJ, squat jump and CMJ, countermovement jump.

Subsequently, all athletes participating in the study were reintegrated to the usual training by continuing with three blocks of dynamic tasks of exercises for 40min with an RPE of 5–6. Specifically, the first block (15min) consisted of the application of attack techniques with hands (four sets of 20 repetitions of attack techniques with straight punches with the front hand, and later with the backhand with a recovery of 3min between series). The second block (15min), consisted of the application of attack techniques with kicks (four sets of 20 “mawashi geri” with the front leg, and then with the back leg with a recovery of 3min between sets). The third block consisted of free combats (15min) with the permanent intervention of the coach to point out technical and tactical aspects. The training sessions finished with stretching (10min; Figure 2).

### Procedures

A week prior the investigation began the athletes completed a familiarization session and EG practiced the HIIT protocol to reduce the learning effect. In addition, both the coach and athletes received an induction on the RPE 0–10 scale. All assessments were scheduled between 9:00 and 11:00AM, completed in the same order, at the same location (gymnasium with wooden floor), with the same sports clothing, and by the same sports science professional before and after the intervention, previously blinded to the intervention. Previously, all participants were instructed to (i) sleep 8h between each assessment session, (ii) not to modify their usual eating and hydration habits during the days before the assessments, and (iii) not to consume caffeinated beverages. The tests were assessed according to exercise intensity in the following order: SJ, CMJ, and T-test. Before the execution of the tests, a general warm-up of ~10min (e.g., submaximal running with a change of direction, 10 vertical and 10 horizontal submaximal jumps) was performed. This was followed by a specific warm-up with potentiation exercises, including stretching, and two submaximal jumping attempts (~5min). The best of the two attempts was considered for performance for each assessment. A 2-min rest interval was performed between each trial, and a 5–10-min rest interval was applied between each test to reduce the effects of fatigue.

### Statistical Analysis

Data analysis was performed with SPSS version 26 for Mac (SPSS Institute, Chicago, IL, United States). Data are presented as mean±SD. Homoscedasticity of variance and normality was checked by Levene’s test and the Shapiro-Wilk test, respectively. The unpaired *t*-test was used to examine for possible gender biases. The interaction of group (inter-subject factor) EG vs. CG and time (intra-subject factor) pre-intervention vs. post-intervention was analyzed by a repeated-measures mixed ANOVA. If significant effects or interactions were observed, the Bonferroni *post hoc* test was applied to adjust for differences between the means of the two groups. For ANOVA outcomes, effect sizes (ES) were calculated using partial eta squared (η^2^_p_). Complementarily, post-intervention changes within and between groups were calculated using Cohen’s d following the classification proposed by Rhea for recreationally trained participants (individuals training consistently for 1–5years; trivial <0.25; small 0.25–0.50; moderate 0.50–1.0; large >1.0; Rhea, 2004). Subsequently, the sample was classified into Rs and NRs using the two-technical error (TE) criterion according to a previously established equation (Bonafiglia et al., 2016). NRs were identified and defined as individuals who were unable to demonstrate an increase or decrease (in favor of beneficial changes) in sport-related fitness that was greater than twice the TE away from zero (Ramirez-Campillo et al., 2018). For the current study, two replicates of all outcomes analyzed were used to calculate TE. A change beyond twice the TE was representative of a high probability (i.e., 12–1 odds) that the observed response was a true physiological adaptation beyond what might be expected as a result of technical and/or biological variability (Ramirez-Campillo et al., 2018). Therefore, the TEs were as follows: [SJ; 3.10 (cm)×2; CMJ, 3.32 (cm)×2; T-test, 0.28 (s)×2]. All assessments showed acceptable reliability coefficient of variation or CV<5% and intraclass correlation or ICC>0.90 (Hopkins, 2000). The level of statistical significance used was set at *p*<0.05.

## Results

No significant differences were reported between both genders in chronological age (*t*=−0.22; *p*=0.08), body mass (*t*=−0.76; *p*=0.46), and stature (*t*=−1.66; *p*=0.13), SJ (*t*=0.29; *p*=0.77), CMJ (*t*=0.81; *p*=0.42), and CODS (*t*=2.20; *p*=0.06).

### Effect and Interaction of the Factors Analyzed

Table 1 presents the summary of the time factor analysis independently in each group and group-by-time interaction for SJ, CMJ, and CODS. Specifically, for SJ no significant effect was reported in the group factor (F_1,8_=1.03; *p*=0.33; *η*^2^_p_=0.11) and time factor (F_1,8_=4.53; *p*=0.06; *η*^2^_p_=0.36). Neither for CMJ in the group factor (F_1,8_=0.15; *p*=0.70; *η*^2^_p_=0.01) and time factor (F_1,8_=0.19; *p*=0.67; *η*^2^_p_=0.02). In addition, the CODS showed no significant effect in the group factor (F_1,8_=0.65; *p*=0.44; *η*^2^_p_=0.07) and time factor (F1,8=0.45; *p*=0.51; *η*^2^_p_=0.05).

**Table 1:** Effects and response rate of high-intensity interval training (HIIT) with specific techniques vs. usual training (*n*=10).

	EG (*n*=5)						CG (*n*=5)						EG vs. CG
	Pre intervention	Post intervention	F_1,8_; *p*; *η*^2^_p_	% change±SD	ES	Rs; %	Pre intervention	Post intervention	F_1,8_; *p*; *η*^2^_p_	% Change±SD	ES	Rs; %	F_1,8_; *p*; *η*^2^_p_
**Outcomes**													
SJ (cm)	26.9±4.5	31±5	3.86; 0.85; 0.32	15.2±11.9	0.91 Moderate	2 (40)	24.8±7.3	26.9±3.1	1.09; 0.32; 0.12	16.2±36.6	0.28 Trivial	1 (20)	0.42; 0.53; 0.05
CMJ (cm)	29.0±5.6	28.1±4.1	0.14; 0.71; 0.17	−1.8±10.1	−0.16 Trivial	0 (0)	27.6±3.7	27.0±7.9	0.59; 0.81; 0.00	2.9±24.2	−0.16 Trivial	1 (20)	0.09; 0.92; 0.01
CODS (s)	12.28±0.68	12.04±0.80	1.60; 0.24; 0.16	−1.7±3.8	−0.35 Small	2 (40)	12.80±1.69	12.86±1.65	0.09; 0.76; 0.01	0.48±2.45	0.03 Trivial	0 (0)	1.23; 0.29; 0.13

*EG, experimental group; CG, control group; % change, changes in means with 90% CI; SD, standard deviation; ES, effect size with 90% CI; Rs, responders; F, value of F; p, value of p; η^2^_p_, partial Eta squared; SJ, squat jump; and CMJ, countermovement jump*.

### Magnitude of Change Based on Inference

Table 1 presents the changes based on inference after the intervention. Particularly, in EG increases in jumping ability were reported for SJ with a *moderate* increase (15.2%; ES=0.91). In contrast, in CG a *trivial* increase in this outcome (16.2%; ES=0.28). For CODS, an increase *small* in performance was reported in EG (−1.7%; ES=−0.35). On the other hand, a *trivial* decreased performance in CG (0.48%; ES=0.03).

On the other hand, for CMJ performance a decrease was reported *trivially* in EG (1.7%% ES=0.35) and CG (0.48% ES=0.03).

### Inter-individual Variability in Response to the HIIT Program

**Figure 3:**
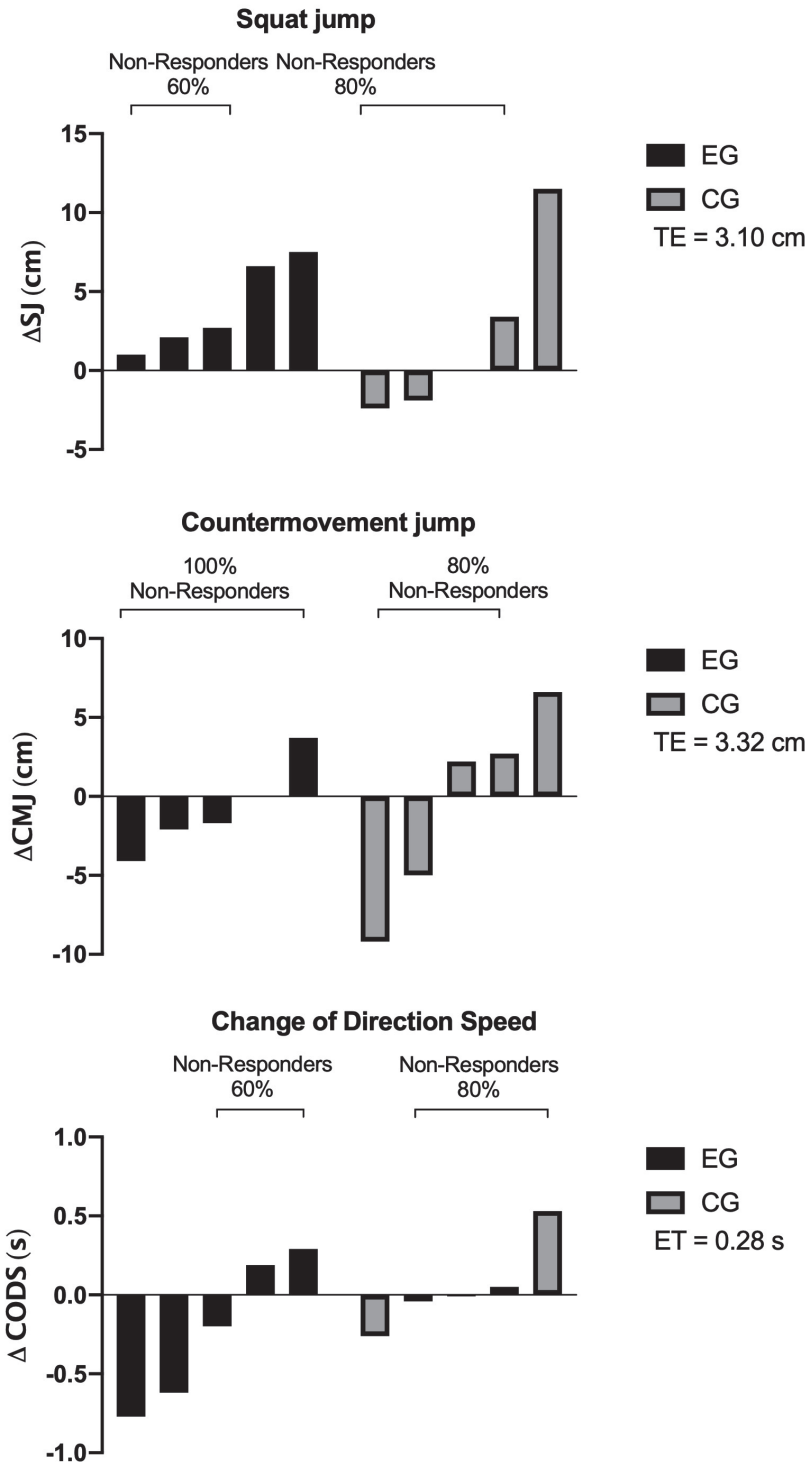
Inter-individual variability analysis of the outcomes analyzed (*n*=10). NRs, non-responders; TE, technical error; Δ, difference post-pre intervention expressed as delta; EG, experimental group; CG, control group; SJ, squat jump; CMJ, countermovement jump; and CODS, change of direction speed.

Figure 3 and Table 1 show the inter-individual variability analysis of jumping ability and CODS in athletes from both groups analyzed. In particular, in EG athlete Rs were reported for SJ and T-test (*n*=2; 40%). Additionaly, for CMJ in the CG (*n*=1; 20%).

On the other hand, in EG for CMJ, 100% of the athletes were classified as NRs.

## Discussion

This study investigated the effect of 4weeks of HIIT with specific techniques and analyzed inter-individual variability (classified in Rs and NRs) on jumping ability and CODS in youth karate athletes. Among the main results, no significant group-by-time interaction effect was found. However, increases in performance in percentage terms and ES in EG were found for CODS and both groups for SJ. At the same time, a trend of higher percentage of Rs athletes in EG vs. CG was observed for SJ and CODS. Consequently, the stated hypothesis was not fulfilled. Indicating that the addition to regular training of a HIIT with specific techniques and based on the temporal structure of combat after 4weeks was not a sufficient stimulus to increase jumping ability and change of direction speed in karate athletes.

### Jumping Ability

Current evidence shows inconsistencies to the effect of HIIT with specific techniques on this physical ability in karate athletes. In this regard, a previous study on HIIT in karate did not document significant effects for SJ and CMJ after comparing a HIIT based on repeated-CMJ vs. repeated-sprints incorporated during the usual training session after 6weeks (Ojeda-Aravena et al., 2019). Although, the authors reported percentage and ES increases for SJ in the repeated-CMJ group (5%; ES=0.30) and repeated-sprints group (8.3%; ES=0.82). Additionally, in another combat sport such as taekwondo, not reported significant increases in CMJ after adding two sessions of simulated combat in different areas sizes (4×4m; 6×6m; and 8×8m) to regular training after 8weeks (Ouergui et al., 2021). On the other hand, in the same sport, Ouergui et al. (2020) documented significant increases for CMJ height in both groups independently after comparing a HIIT with repeated-sprints vs. technical-specific efforts (three sets of 10 repetitions of 6s of repeated kicks with 10s of rest between repetitions and 3min of recovery between sets) after 4weeks (Ouergui et al., 2020).

According to the above, through the results analyzed and given the heterogeneity of the HIIT protocols applied, it is still not possible to be conclusive about the increase in performance in this physical ability in karate. In this sense, the lack of volume of training load applied may have influenced the results. In this regard, studies in combat sports such Taekwondo, that report significant increases in jumping ability add independent training sessions (Ouergui et al., 2020, 2021). Furthermore, the different motor patterns used during HIIT (running, jumping, and specific techniques) involve different muscle tension for the lower limbs and potentially different adaptations. The above, considering the growing evidence in other collective sports such as ice hockey (Kinnunen et al., 2019), highly trained running athletes (Kohn et al., 2011), and recreationally active individuals (Schaun et al., 2019; Moghaddam et al., 2020) who primarily use HIIT based on running. In these studies, neuromuscular (improvement of lower body muscular power, increase of the force-velocity curve and decreased maximal voluntary contraction times), histological (increases in type 2 fiber pool, increases in muscle cross-sectional area), and biochemical (increases in lactate dehydrogenase and decreased lactate levels; Kohn et al., 2011; Kinnunen et al., 2019; Schaun et al., 2019; Moghaddam et al., 2020) adaptations are reported.

Based on the current background, it would be necessary to increase the weekly training volume, either with a higher number of rounds during the training session, by adding independent HIIT sessions or by increasing the training weeks. In addition to HIIT, other specific training strategies such as high-intensity functional training, plyometric training or speed-based training could be applied in order to optimize the dynamic strength components (Loturco et al., 2014; Neto and Kennedy, 2019; Franchini, 2020; Quinzi et al., 2020).

### Change of Direction Speed

According to the CODS results, an increase in percentage and ES (−1.7%; −0.35, respectively) was observed post-intervention. About this, the evidence regarding the improvement of CODS using HIIT in karate athletes is still controversial. In this regard, for example, previously in this sport, percentage and ES increases (−11.6%; ES=1.20) are observed, although without significant decreases in the performance of this ability after HIIT intervention based on repeated-CMJ vs. repeated-sprints (Ojeda-Aravena et al., 2019). On the other hand, in taekwondo, significant increases (*p*=0.04) in this ability are documented in favor of the group that performed simulated bouts in the 4×4m vs. 6×6m and 8×8m area size after 8weeks (Ouergui et al., 2021). In addition, this same group of researchers in youth taekwondo athletes reported significantly greater performance in the HIIT with specific-techniques vs. HIIT with repeated-sprints (*p*<0.01) after 4weeks of training (Ouergui et al., 2020).

However, despite the growing positive evidence of HIIT on this physical ability, it is still not possible to state this with certainty, considering the disparity of protocols and the number of athletes analyzed. Also, the lack of specific neuromuscular stress between HIIT and CODS has likely influenced the results obtained. In this sense, it may be that the lack of accelerations and decelerations in the motor patterns used influenced the observed response. In this regard, it is important to emphasize that in acute terms the evidence shows that muscle power expressed indirectly through jumping ability and including indirect eccentric indexes, in this sport is significantly related to and influences CODS performance (Herrera-Valenzuela et al., 2020; Ojeda-Aravena et al., 2021a). Furthermore, these results are consistent when examining the relationship between jumping ability and specific CODS in male youth karate athletes (Herrera-Valenzuela et al., 2020).

Another aspect that may have affected the analyzed results is the phenomenon of interference about muscle hypertrophy and/or power or force rate development adaptations resulting from concurrent training (strength and endurance) performed during the same session or as part of the training program (Wilson et al., 2012; Coffey and Hawley, 2017; Fyfe and Loenneke, 2018; Neto and Kennedy, 2019). However, this phenomenon is currently debated and associated factors (including exercise volume, intensity, and nutritional status, among others) must be taken into account (Neto and Kennedy, 2019). Evidence points out that such an effect could also depend on the participants’ general fitness level, their training experience, and the frequency of sessions in a week (Fyfe and Loenneke, 2018). On the other hand, HIIT has been shown to reduce the phenomenon of concurrent training interference (Methenitis, 2018). Additionaly, it is important to mention that this study did not apply strength training with external loads, therefore, it is pertinent to question whether bodyweight training could be sufficient to generate this phenomenon or whether it is due to the aforementioned factors.

### Inter-individual Variability in Response to the HIIT Program

Another purpose was to analyze the athletes inter-individual variability. Among the main results, Rs were reported for the two groups in SJ and only for the T-test in EG. Meanwhile, athletes’ NRs were reported for all the analyzed outcomes. These results are similar to those reported recently in taekwondo athletes after 4weeks of HIIT with specific-techniques (Ojeda-Aravena et al., 2021b). In this study, the authors documented Rs for SJ (*n*=2) and CODS (*n*=3; Ojeda-Aravena et al., 2021a). In another combat sport such as boxing, recently the authors Herrera-Valenzuela et al. (2021) interestingly documented after the application of a HIIT with specific-techniques after 4weeks a higher proportion of athletes Rs in outcomes related to specific actions and performance of bipodal and unipodal CMJ of both limbs (Herrera-Valenzuela et al., 2021).

Accordingly, the inter-individual variability of observed responses to training, including HIIT, according to Walsh et al. (2020) is a combination of (i) individual responses to perseverative exercise training (subject-training interaction), (ii) day-to-day biological variation, and technical error (random variation), and (iii) physiological responses associated with behavioral/maturational changes not attributable to exercise (e.g. within-person variability; Walsh et al., 2020). This includes genetic (Mann et al., 2014; Sparks, 2017; Bonafiglia et al., 2020; Del Coso et al., 2020), climatic (Corbett et al., 2018), cognitive (Atkinson and Batterham, 2015), stress and sleep status (Mann et al., 2014), gender, age, time of day variation (Mann et al., 2014; Sparks, 2017), training status (Pickering and Kiely, 2019), physiological (Williamson et al., 2017; Atkinson et al., 2019), and statistical (Swinton et al., 2018; Chrzanowski-Smith et al., 2020).

### Limitations

However, it is important to mention that the results should be analyzed for their merit, as they could be influenced by (i) the small sample size, (ii) the menstrual cycle of females (Schmitz et al., 2020); (iii) the lack of neuromuscular stress applied; and (iv) the homogeneity of the athletes according to their biological age. Nevertheless, considering the above, the incorporation of HIIT with specific-techniques in combat sports fitness is an early-stage research topic in applied sports science reflected in growing evidence (Franchini et al., 2016, 2017; Kamandulis et al., 2018; Ouergui et al., 2020, 2021; Herrera-Valenzuela et al., 2021; Ojeda-Aravena et al., 2021a). In this sense, future research could use a greater number and experience level of athletes and verify the results by gender. Also, could verify the physiological and neuromuscular effect of HIIT protocols with specific-techniques, in addition to verifying the efficient interval for this sport.

### Highlights

Although it requires further study, the incorporation of HIIT protocols with specific-techniques and using the time structure of combat could be an alternative as part of the training session during inter-competitive periods (e.g., during a shock microcycle) due to the limited time available to athletes to cope with the demands of this period. In addition, these HIIT protocols can be performed in reduced places. In turn, coaches could use inter-individual response analysis as a practical monitoring tool to follow the training progress of each athlete.

## Conclusion

In conclusion, the addition to regular training of a HIIT protocol with specific techniques and based on the temporal structure of combat after 4weeks was not a sufficient stimulus to increase jumping ability and change of direction speed in karate athletes.

## Data Availability Statement

The raw data supporting the conclusions of this article will be made available by the authors, without undue reservation.

## Ethics Statement

The studies involving human participants were reviewed and approved by the Ethics Committee of Universidad Autónoma (Code: 080–18). Written informed consent to participate in this study was provided by the participants’ legal guardian/next of kin.

## Author Contributions

All authors listed have made a substantial, direct and intellectual contribution to the work, and approved it for publication.

## Conflict of Interest

The authors declare that the research was conducted in the absence of any commercial or financial relationships that could be construed as a potential conflict of interest.

## Publisher’s Note

All claims expressed in this article are solely those of the authors and do not necessarily represent those of their affiliated organizations, or those of the publisher, the editors and the reviewers. Any product that may be evaluated in this article, or claim that may be made by its manufacturer, is not guaranteed or endorsed by the publisher.
